# Overexpression of EMT-related transcription factors SNAI1 and ZEB1 is associated with more aggressive clinicopathological features of pancreatic cancer

**DOI:** 10.1371/journal.pone.0339964

**Published:** 2026-01-02

**Authors:** Kestutis Urbonas, Gabija Stachneviciute, Aldona Jasukaitiene, Povilas Ignatavicius, Zilvinas Dambrauskas, Antanas Gulbinas, Giedrius Barauskas

**Affiliations:** 1 Department of Surgery, Lithuanian University of Health Sciences, Kaunas, Lithuania; 2 Institute for Digestive Research, Laboratory of Surgical Gastroenterology, Lithuanian University of Health Sciences, Kaunas, Lithuania; Longgang Otorhinolaryngology Hospital & Shenzhen Key Laboratory of Otorhinolaryngology, Shenzhen Institute of Otorhinolaryngology, CHINA

## Abstract

**Background/Objectives:**

Pancreatic ductal adenocarcinoma (PDAC) is one of the most fatal malignancies due to its aggressive nature and resistance to therapy. The epithelial-mesenchymal transition (EMT) drives cancer progression, regulated by transcription factors (TFs) such as SNAI1, SNAI2, ZEB1, ZEB2, and TWIST. This study evaluates EMT-TF expression in PDAC and its clinical relevance.

**Methods:**

PDAC tissues from 45 patients were analyzed using qRT-PCR and Western blot. Clinical features and survival outcomes were statistically examined for correlations with EMT-TF levels.

**Results:**

mRNA levels of SNAI1 (16.4-fold, p = 0.02), SNAI2 (21.8-fold, p = 0.028), ZEB1 (17.2-fold, p = 0.037) were significantly elevated in PDAC tissues compared to healthy controls. TWIST showed 3.75-fold increase in PDAC tissue; however, this elevation was not significant (p = 0.124). Corresponding protein-level increases were observed for Snail1/Slug and Zeb1. High SNAI1 expression correlated with peripancreatic invasion (p = 0.026) while ZEB1 overexpression was significantly associated with shorter survival (15.2 vs. 33.3 months, p = 0.037) and remained an independent prognostic factor in multivariate analysis. ZEB2 mRNA was reduced, however, protein levels were elevated. TWIST mRNA overexpression was not reflected at the protein level.

**Conclusions:**

Overexpression of EMT transcription factors SNAI1 and ZEB1 reflect more aggressive histopathological patterns of PDAC. The strong correlation of SNAI1 expression with the expression of other EMT-TFs highlights its’ role in peripancreatic invasion, as well as impact on overall survival and may serve as an argument defining the leading role of SNAI1 in this context.

## Introduction

Pancreatic ductal adenocarcinoma (PDAC) remains one of the most fatal tumors with 5-years survival rate of a 7–11% [1]. Majority of patients are diagnosed in advanced stages of the disease and only patients undergoing curative resection for pancreatic cancer have 15–20% overall years survival rate [2]. Improved understanding of genetics, epigenetics, metabolomics and inter-cellular interplay still does not result in clinically significant benefits for patients with pancreatic adenocarcinoma.

The aggressiveness of the tumor is highly associated with epithelial-mesenchymal transition (EMT) contributing to chemoresistance, metastasis, and recurrence [3]. Epithelial to mesenchymal transition is a reversible biological process in which epithelial cells lose their unique features of apicobasal polarity and usually is understood as epithelial-mesenchymal plasticity [4]. This physiological event is observed during embryogenesis (type I), but it also happens in fibrosis (type II) and cancer metastasis (type III) [5]. EMT is characterized by the loss of cell-to-cell junctions and apico-basolateral polarity, resulting in the formation of migratory mesenchymal cells with invasive properties. Throughout the EMT phenomena cancer cells acquire the ability to migrate, resist therapeutic agents, escape immunity, and form new colonies. EMT is regulated at multiple levels: transcriptional, translational, protein stability and epigenetic. EMT inducers lead to expression and functional activation of core regulators, including among others three major groups of EMT-activating transcription factors (EMT-TFs): the Snail family of the zinc-finger transcription factors Snail/Slug encodeded by SNAI1 and SNAI2, the zinc-finger E-box binding homeobox (ZEB) family of transcription factors ZEB1/ZEB2, and the Twist family of basic helix-loop-helix (bHLH) transcription factors TWIST1/TWIST2 [6].

Recent studies and experimental models have disclosed that EMT in pancreatic cancer is mostly triggered by Zeb1, promoting cell plasticity and playing a key role in early dissemination of PDAC [7–9]. However, neither of the known EMT promoters were shown to have a significant impact on survival of PDAC patients. Snail family seems to stand in the prime chain activating EMT process and plays a major role in early dissemination and progression of other types of cancer like gastric, breast or colon [10–12].

The knowledge on how EMT process impacts the survival of PDAC patients is limited. There are studies showing that EMT-TF influence early dissemination, local recurrence and chemoresistance of a PDAC; however, neither of EMT-TF’s is known to be superior. The aim of this study was to investigate the expression patterns and the clinical relevance of EMT-TFs in PDAC patients undergoing curative resection.

## Materials and methods

### Study population and tissue sample collection

PDAC tissue was obtained from 45 patients who underwent pancreatoduodenectomy at the tertiary reference center between 2011 and 2020. All patients were diagnosed with PDAC in the head of the pancreas and no history of other malignancies. The diagnosis was confirmed by the routine histological evaluation of the surgical specimens. Tumor staging was performed according to the AJCC 7th edition (2010), which was applicable during the study period (2011–2017). Peripancreatic invasion was defined histologically as infiltration of peripancreatic fat tissue beyond the pancreatic parenchyma. All patients were referred for adjuvant gemcitabine-based chemotherapy according to national treatment guidelines of the study period. There were no patients with neoadjuvantchemotherapy included in this study. Healthy pancreatic tissue was harvested from six multi-organ donors who had no previous history of cancer. The median age of the donors was 52 years (3 male, 3 female), and all causes of death were non-malignant (traumatic brain injury or intracranial hemorrhage).For qRT-PCR analysis freshly removed tissue samples were placed in RNALater (Ambion; Huntingdon, United Kingdom), whereas tissues for protein extraction were snap frozen in liquid nitrogen in the operating room upon surgical removal and maintained at −80 °C until use. Ethical approval was issued by the Ethics Committee of the Lithuanian University of Health Sciences (No. BE-2–10). Written consent for the use of tissues specimens and follow up data for research purposes was obtained from all the patients or their legal representatives. Patients were followed until death or last contact. Because the primary cause of death could not always be reliably determined, only overall survival (OS) was analyzed.

### RNA extraction and reverse transcription PCR

Total RNA extraction was performed from tissues and using PureLink RNA easy kit (Ambion) and TRI reagents (Zymo), according to the manufacturer’s protocol without DNAse treatment. Purified RNA was quantified and assessed for purity by UV spectrophotometry (NanoDrop). Complementary DNA (cDNA) was generated from 2 μg of RNA with High-Capacity RNA-to-cDNA Kit (Applied Biosystems). The amplification of specific RNA was performed in a 20 μL reaction mixture containing 2 μL of cDNA template, 1 × PCR master mix and the primers. Quantitative reverse transcription-PCR (qRT-PCR) analysis was performed using ABI 7500 fast Real-Time PCR system (Applied Biosystem). For normalization, GAPDH housekeeping gene was used. Relative quantification was performed using the 2^- ∆∆Ct^ method.

### Western blot assay

Whole cells were lysed using the RIPA lysis buffer with protease inhibitors (Roche) and centrifuged at 10000 × g for 10 min. The supernatants were assayed for protein concentration with a BCA protein assay kit (Thermo Scientific). Protein samples were heated at 97°C for 5 min before loading and 50 μg of the samples were subjected to 4%−12% sodium dodecyl sulfate-polyacrylamide gel electrophoresis (SDS-PAGE), and transferred to poly-vinylidene fluoride (PVDF) membranes for 50 min at 20 V. The membranes were blocked with a blocking buffer (Invitrogen) for 30 min at room temperature and incubated overnight at 4°C with primary antibodies: 1:1000 mouse monoclonal anti-Snail+SLUG from Abcam (ab180714), 1:5000 rabbit monoclonal anti-Zeb1 from Abcam (ab124512), 1:1000 rabbit monoclonal anti-Zeb2 from Abcam (ab223688), 1:1000 rabbit monoclonal anti-TWIST (ab175430), and 1:10000 mouse monoclonal anti- GAPDH from Ambion (AM4300). The membranes were washed and incubated with the appropriate peroxidase-conjugated secondary antibody (Invitrogen; anti-mouse or anti-rabbit) for 30 min, washed and incubated with a chemiluminescence substrate/detection kit (Invitrogen). Results were analyzed with an automated documenting system (Biorad).

### Statistical analysis

Statistical analysis was performed using GraphPad (version 8.2; GraphPad Software Inc., La Jolla, CA, USA) software. The data are presented as mean ± SD of three or more independent experiments. A nonparametric Mann–Whitney test was used for comparison between groups. Multivariate analysis was performed using the Cox proportional regression model. Spearman’s rank correlation test was used to determine correletion among different EMT TFs. Statistical significance was defined as p < 0.05.

## Results

### Characteristics of the patients

The median patients’ age was 68 (range 44–87). Histopathological evaluation revealed that majority of patients had T3 (64.4%) and well to moderate differentiated G1-G2 (60%) tumors. Regional metastatic lymph-nodes (N1) and peripancreatic invasion were detected in 68.9% of cases. Microvascular invasion (V1) was present in 66.7% and perineural invasion in 64.4% of cases. The median follow-up time was 96.8 months, and the median survival was 30.2 months. All descriptive data of the study group are presented in Table 1.

**Table 1:** Characteristics of patients after pancreatoduodenectomy for PDAC.

Characteristic of PDAC patients	n (%)
**T stage**	
T1	5 (11.1)
T2	5 (11.1)
T3	29 (64.4)
T4	6 (13.4)
**N status**	
N0	14 (31.1)
N1	31 (68.9)
**Differentiation grade**	
Well to Moderate (G1-G2)	27 (60)
Poor (G3-G4)	18 (40)
**Perineural invasion**	
Yes	29 (64.4)
No	16 (35.6)
**Lymphatic invasion**	
Yes	31 (68.9)
No	14 (31.1)
**Microvascular invasion**	
Yes	30 (66.7)
No	15 (32.3)
**Peripancreatic invasion**	
Yes	31 (68.9)
No	14 (31.1)
**R status**	
R0	34 (75.5)
R1	11(24.5)

PDAC – pancreatic ductal adenocarcinoma

### EMT transcription factors show differential overexpression in PDAC compared to healthy pancreatic tissue

**Fig 1:**
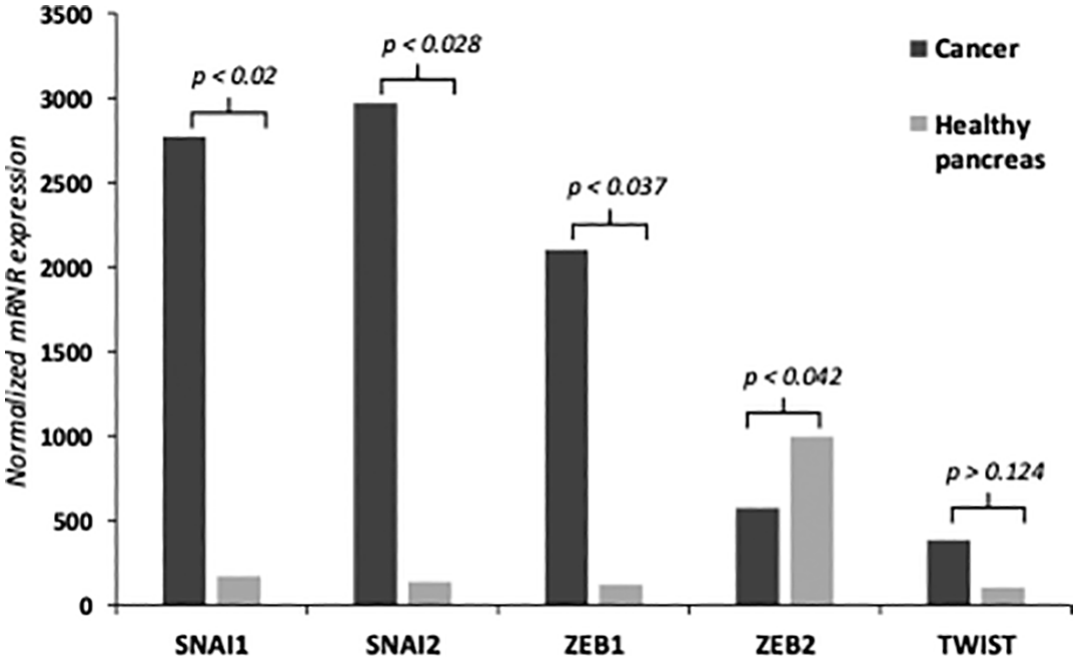
mRNA expression of EMT-TFs. qRT-PCR analysis showed significantly elevated mRNA levels of SNAI1, SNAI2, ZEB1, and TWIST in PDAC tissue, while ZEB2 expression was higher in normal pancreatic tissue.

**Fig 2:**
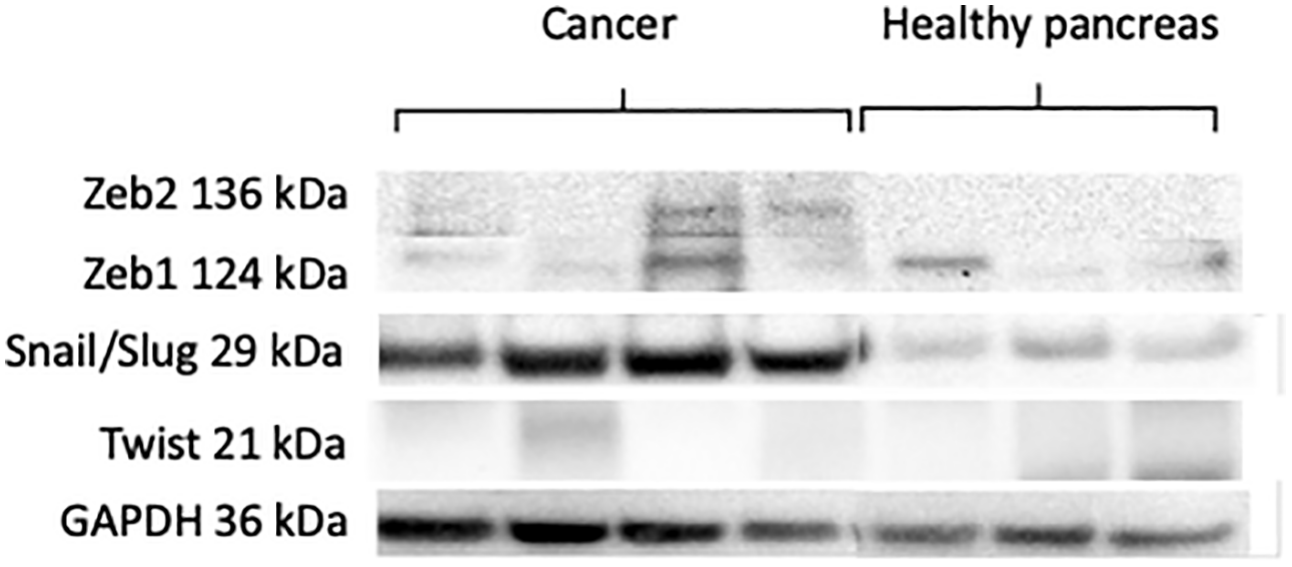
Protein expression of EMT transcription factors. Western blot analysis confirmed increased protein levels of Snail1/Slug, Zeb1, and Zeb2 in PDAC, whereas Twist protein expression was undetectable despite its elevated mRNA levels.

The comparison of EMT-TFs expression levels between PDAC and healthy pancreatic tissue revealed significantly elevated mRNA levels of SNAI1, SNAI2, ZEB1 and TWIST in pancreatic cancer tissue, while ZEB2 expression was higher in normal pancreatic tissue (p < 0.05) (Fig 1). Furthermore, Western blot assay revealed increased protein levels of Snail1/Slug, Zeb1 and Zeb2 compared to normal pancreatic tissue while Twist expression was unnoticeable (Fig 2). SNAI1, SNAl2 and ZEB1 were overexpressed at mRNA level (16.4-fold, 21.8-fold and 17.2-fold respectively) and protein level in cancer tissue as compared to normal pancreatic tissue. ZEB2 mRNA expression was 1.7-fold lower in cancer tissue as compared to normal pancreatic tissue, but higher protein amounts were determined in Western blot analysis. TWIST mRNA expression was 3.75-fold higher in PDAC patients, but we could not determinate any protein expression in Western blot analysis (Figs 1 and 2).

### SNAI1 overexpression correlates with elevated expression of other EMT-TFs and peripancreatic tumor invasion in PDAC

SNAI1 overexpression demonstrated a strong correlation with the high expression levels of SNAI2, ZEB1, ZEB2, and TWIST on mRNA level (Table 2).

**Table 2:** Correlation analysis (Spearman’s rho).

		SNAI1	SNAI2	ZEB1	ZEB2	TWIST
**SNAI1**	Correlation Coefficient	1.000	.929	.815**	.317*	.374*
	Sig. (2-tailed)	.	<.001	<.001	.036	.013
**SNAI2**	Correlation Coefficient	.929	1.000	.837	.317	.417
	Sig. (2-tailed)	<.001	.	<.001	.036	0.005
**ZEB1**	Correlation Coefficient	.815**	.837	1.000	.246	.312*
	Sig. (2-tailed)	<.001	<0.01	.	.107	.039
**ZEB2**	Correlation Coefficient	.317*	.317	.246	1.000	.628**
	Sig. (2-tailed)	.036	.036	.107	.	<.001
**TWIST**	Correlation Coefficient	.374*	.417	.312*	.628**	1.000
	Sig. (2-tailed)	.013	.005	.039	<.001	.

**. Correlation is significant at the 0.01 level (2-tailed).

*. Correlation is significant at the 0.05 level (2-tailed).

The correlation between individual EMT-TFs and histopathological variables revealed that SNAI1 overexpression was significantly associated with higher rate of peripancreatic invasion (p = 0.026, as shown in Table 3). In contrast, the remaining EMT-TFs did not show any significant correlation with histopathological variables.

**Table 3:** EMT TF’s correlation analysis with histopathological features of PDAC (*p*-value).

	T stage		N status		Peripancreatic invasion		Microvascular invasion		Perineural invasion	
*χ*^2^ value	p-value	*χ*^2^ value	p-value	*χ*^2^ value	p-value	*χ*^2^ value	p-value	*χ*^2^ value	p-value
**SNAI1**	0.007	0.62	4.31	0.11	5.13	0.026*	1.13	0.23	0.18	0.46
**SNAI2**	1.8	0.17	0.48	0.49	0.64	0.8	0.36	0.55	1.2	0.27
**ZEB1**	1.48	0.29	2.7	0.25	0.11	0.54	0.4	0.47	0.025	0.69
**ZEB2**	0.9	0.29	1.39	0.49	0.31	0.41	0.00	0.63	0.09	0.51
**TWIST**	0.4	0.4	2.39	0.3	0.014	0.58	0.00	0.63	0.13	0.48

*. Correlation is significant at the 0.05 level (2-tailed).

### Impact of clinicopathological parameters and EMT TF’s expression on survival of PDAC patients

**Fig 3:**
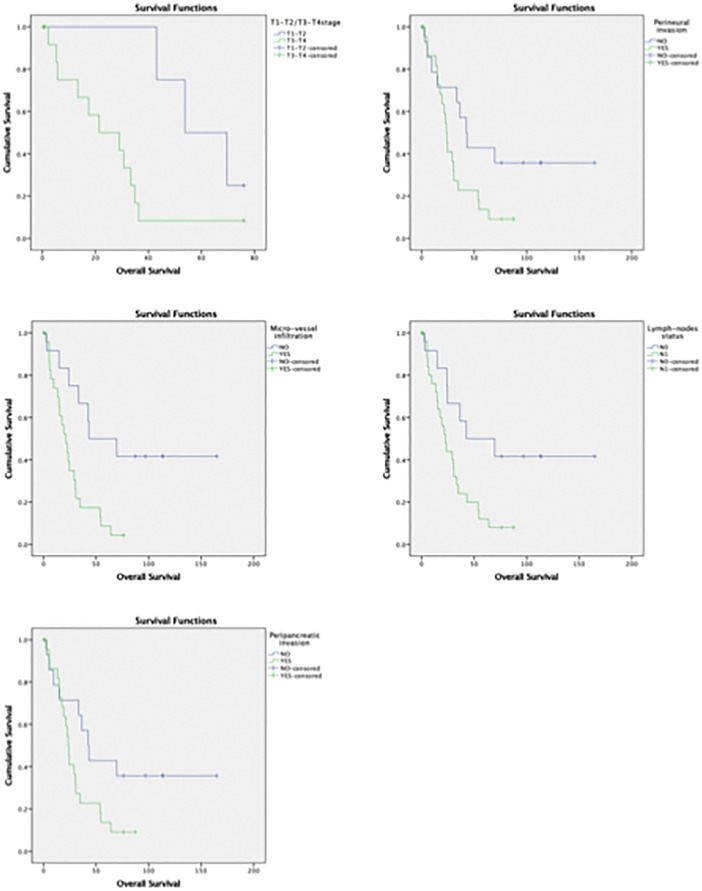
Survival analysis based on PDAC histopathological features. Kaplan-Meier survival curves were calculated to compare overall survival in PDAC patients stratified by key histopathological variables. Significant differences in survival were observed between groups with advanced tumor stages (median survival: 21.3 vs. 53.7 months), perineural invasion (23.3 vs. 42.4 months), microvascular invasion (21.3 vs. 43 months), nodal involvement (22.5 vs. 42.3 months), and peripancreatic invasion (23.4 vs. 42.4 months), (p < 0.05 for all comparisons).

Univariate survival analysis was performed including clinical and pathological variables of PDAC patients. Analysis revealed that tumor size, positive lymph-nodes, peripancreatic invasion, perineural spread and microvascular invasion had a significant impact on overall survival (Table 4, Fig 3). Analysis of mRNA expression levels of EMT genes revealed that the largest area under the curve (AUC) was consistently observed when using the median expression level as a cutoff for all genes. Since the median AUC was the highest compared to Q1 and Q3 across all subgroups, we selected the median mRNA expression level as the threshold for subsequent survival analysis to ensure robust and balanced comparisons.

**Table 4:** Univariate analysis of pathological variables of PDAC patients (log-rank).

Variable	P value	HR
**T stage** T1-T2/T3-T4	**0.04**	**1.92**
**N status** N0/N1	**0.016**	**2.54**
**Tumor differentiation** Well to Moderate/Poor	0.45	1.6
**Peripancreatic invasion** YES/NO	**0.029**	**2.54**
**Perineural invasion** YES/NO	**0.048**	**2.21**
**Micro-vessel infiltration** YES/NO	**0.015**	**3.46**
**R status** R0/R1	0.065	2.32

**Fig 4:**
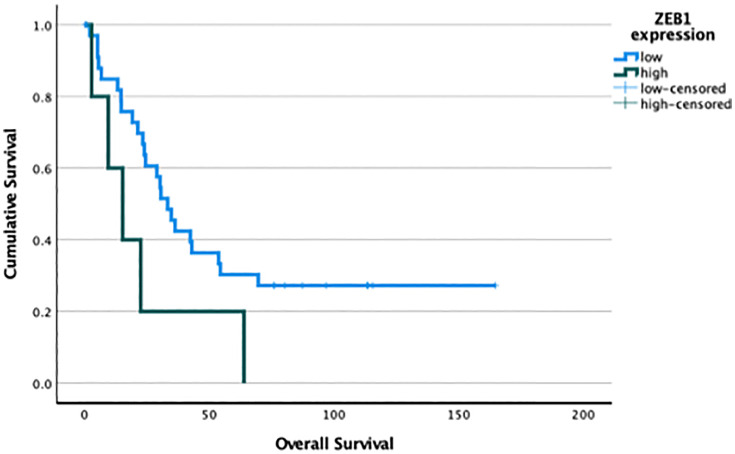
Survival analysis based on ZEB1 expression in PDAC. Kaplan-Meier survival curves comparing overall survival in PDAC patients. Significant differences in survival were observed between groups with high and low ZEB1 mRNA expression (15.2 vs. 33.3 months), (p < 0.05 for all comparisons).

SNAI1, SNAI2, ZEB1, ZEB2, and TWIST median expression values were chosen to categorize PDAC patients into low and high expressing subgroups (Table 5). The group of patients with high SNAI1 expression had a median survival of 22.5 months, whereas the group with low expression had a median survival of 34.7 months (HR 1.53, 95% CI 18.5–41.99, p-0.23). However, the difference between the groups was not statistically significant. We evaluated the diagnostic performance of a model using the ROC curve analysis, which resulted in an AUC of 0.63 for SNAI1. This suggests a modest ability to differentiate between the two classes, although it was not statistically significant (95% CI 0.463–0.798, p-0.124), indicating variability in the model’s performance. ZEB1 overexpression was associated with poor overall survival. In PDAC patients with high expression of ZEB1 median survival was 15.2 months as compared to 33.3 months in patients with low expression (p = 0.037, Fig 4). SNAI2*,* ZEB2 and TWIST mRNA expressions had no impact on PDAC patients’ survival in univariate analysis.

**Table 5:** Impact of High and Low mRNA expression of EMT TFs’ on patients’ survival: A Univariate analysis (Log-Rank).

Variable	P value	HR
**SNAI1** High/low	0.20	1.53
**SNAI2** High/low	0.28	1.44
**ZEB1** High/low	**0.037**	**2.41**
**ZEB2** High/low	0.82	0.98
**TWIST** High/low	0.65	1.18

The Cox regression multivariate analysis was carried out including all the EMT TF’s. The results highlighted ZEB1 as the most important independent variable for survival (HR 2.41, 95% CI 0.91–6.43, p-0.041). No other EMT TF’s were significant. Results are presented in Table 6.

**Table 6:** Cox regression analysis of EMT TF’s.

Variable	p value	HR	95% CI
**SNAI1**	0.46	1.53	0.6–3.02
**SNAI2**	0.83	0.88	0.26–2.93
**ZEB1**	**0.041**	**2.41**	**0.91 - 6.43**
**ZEB2**	0.4	0.71	0.33–1.57
**TWIST**	0.98	0.99	0.33–2.98

In the multivariate Cox regression analysis of pathological variables T stage (HR 5.09, 95% CI 1.51–17.14, p – 0.009) and micro-vessel infiltration (HR 5.79, 95% CI 1.25–26.83, p – 0.025) remained significant independent prognostic factors for overall survival, while other parameters did not reach statistical significance (Table 7).

**Table 7:** Cox regression analysis of pathological variables of PDAC patients.

Variable	p value	HR	95% CI
**T stage**	**0.009**	**5.09**	**1.51–17.14**
**N status**	0.71	1.39	0.23 - 8.23
**Tumor differentiation**	0.09	1.95	0.89 - 4.26
**Peripancreatic invasion**	0.26	0.38	0.07 - 2.03
**Perineural invasion**	0.08	0.3	0.08 - 1.16
**Micro-vessel infiltration**	**0.025**	**5.79**	**1.25 - 26.83**
**R status**	0.14	2.19	0.76 - 6.3

A combined multivariate Cox regression model including all pathological variables and EMT-TF expression markers was performed. Only T stage remained an independent predictor of overall survival (HR 24.47, 95% CI 4.46–134.28; p < 0.001). The loss of significance of ZEB1 and other EMT-TFs in the combined model likely reflects overlapping prognostic information, as EMT activation may contribute to tumor progression features already captured by pathological staging. These findings suggest that while molecular factors like ZEB1 are biologically highly relevant, their prognostic impact in clinical setting might well be mediated and reflected through established clinicopathological characteristics (i.e., TNM status, perineural and/or vascular invasion, etc).

## Discussion

The plasticity of cancer cells leads to the adaptation to the microenvironment providing the capabilities to survive and invade the surrounding tissues and gaining the ability to migrate and form distant metastases. Accumulating evidence indicates that activation of EMT confers chemoresistance [13,14].

The overexpression of EMT transcription factors in pancreatic cancer exerts several functional consequences, contributing to disease progression. Our data aligns with previous research that EMT TF’s are overexpressed in pancreatic cancer tissue as compared with healthy pancreas. These factors promote invasiveness, motility, and resistance to apoptosis, all of which are hallmarks of aggressive cancer. Research by Bhattacharjee S et al. showed that SNAI, when overexpressed, induced the migration and invasion of pancreatic cancer cells by repressing E-cadherin expression [15]. Such findings underscore the pivotal role of EMT transcription factors in shaping the malignant phenotype of PDAC. In concordance a study of Zheng et al. found that elevated SNAI expression was independent predictor of poor prognosis in patients with PDAC [16].

Furthermore, the survival benefit depends on microscopic clearance of the resection margin. Two large meta-analyses have demonstrated that patients with microscopic margin clearance (R0 status) showed significantly better survival than patients with microscopic residual tumor (R1 status) [17]. However, Esposito et al. demonstrated the majority of resections should be classified as R1 mostly due to peripancreatic invasion in posterior and medial resection margins that cannot be surgically managed other way [18].

Our multivariate Cox regression analysis, found that T stage and micro-vessel infiltration were independent prognostic factors for overall survival. These findings goes in concordace with the paper from S.Hong emphasisng the importance of microvascular invasion [19]. However, N status, perineural invasion, and peripancreatic invasion did not reach statistical significance in multivariate analysis. These findings indicate that tumor aggressiveness and vascular involvement play dominant roles in predicting patient outcomes. Importantly, the association between SNAI1 expression and peripancreatic invasion remained significant even when adjusted for these clinicopathological factors, supporting the hypothesis that SNAI1 overexpression directly contributes to local tumor invasiveness rather than merely reflecting more advanced disease.

Our data supports the idea that overexpression of SNAI1 may be associated with more invasive features of the pancreatic cancer resulting in peripancreatic invasion. The role of ZEB1 promoting EMT has significant implication for disease progression, as well as SNAI1 – ZEB1 involvement in EMT is well defined and is linked to the acquisition of an aggressive phenotype in PADC cells, including enhanced invasiveness, which is vital for metastatic spread [20]. Furthermore, ZEB1 is associated with the regulation of genes governing cell motility and invasion, such as matrix metalloproteinases and integrins, which are critical factors in tumor invasion [21]. On the other hand, ZEB1 and elevated expression of Vimentin represents a critical aspect of the EMT process. Numerous studies have demonstrated ZEB1 directly regulates the expression of Vimentin directly binding to the promoter region of Vimentin and inducing its transcription, resulting in the acquisition of a mesenchymal phenotype and enhancing tumor cell invasiveness [22]. The clinical relevance of ZEB1 in PDAC patients is substantial and recognized in the complex landscape of pancreatic cancer. The study by Wellner U et al. demonstrated ZEB1 contribution to the pancreatic cancer stem cell hypothesis supporting the idea of local and distant dissemination of the pancreatic cancer [9]. Our study found that ZEB1 is overexpressed at both the mRNA and protein levels in pancreatic cancer tissue, whereas ZEB2 was characterized by lower mRNA and higher protein levels. However, only ZEB1 was associated with poor clinical outcomes.

Jen-Jung P. et al reported the direct regulation of the stemness gene Bmi1 by TWIST. TWIST and BMI1 act cooperatively to repress E-cadherin leading to the induction of EMT and stem-like properties of cancer cells. The evidence provides crucial link between acquisition of metastatic traits and tumor initiating mesenchymal shift in cancer cells undergoing EMT process [16]. On the contrary, our research did not find any correlation between TWIST overexpression and histopathological features of the pancreatic cancer. Our results showed TWIST mRNA overexpression in pancreatic cancer tissue. The impaired protein detection may be due to the ubiquitin-proteasome pathway and/or technical limitations, such as potential dysfunction or insufficient sensitivity of the anti-TWIST antibody used. However, this does not exclude the role of TWIST in the EMT process. Regarding ZEB2, mRNA expression was decreased in tumor tissue while protein levels were elevated. This discrepancy may be explained by post-transcriptional regulation (e.g., microRNAs stabilizing or repressing translation), differences in protein half-life, or translational control mechanisms that allow for higher protein accumulation despite lower transcript abundance.

It is established that SNAI1 can drive the expression of other EMT transcription factors, including ZEB1, TWIST and SNAI2. In particular, SNAI1 can bind to the promoter regions of ZEB1 and activate its transcription, contributing to the downregulation of E-cadherin and promoting the EMT process, which is associated with tumor progression and metastasis. The interaction between SNAI1 and ZEB1 is part of a complex network of transcription factors that regulate cell plasticity during cancer progression, and it is commonly observed in various cancers. Thus, SNAI1 may influence the expression of other EMT-related transcription factors and directly interact with ZEB1 to drive the EMT phenotype [23–25]. Our study emphasizes the critical impact of ZEB1 and SNAI1 overexpression on survival rates in pancreatic cancer, primarily due to their roles in facilitating peripancreatic invasion. We found a strong correlation between SNAI1 expression and increased peripancreatic invasion, highlighting SNAI1’s pivotal role in enhancing tumor aggressiveness. Additionally, we observed that ZEB1 overexpression is contingent upon the overexpression of SNAI1, indicating a regulatory cascade where SNAI1 enhances the expression of ZEB1, further promoting invasive and metastatic behavior of pancreatic cancer cells.

Comparing our findings to the Koster et al. AMC R2 database, expression levels of SNAI1, SNAI2, ZEB1, ZEB2 and TWIST were found to be significantly associated with overall survival [26]. Specifically, both high and low expression patterns of these EMT transcription factors correlated with poor patient outcomes. Notably, in one of the largest publicly available cohorts within the R2 Genomics platform, our observations were inconcordance: ZEB1 expression demonstrated similar survival trends as seen in our cohort, reinforcing its prognostic value. Furthermore, patients with high SNAI1 expression have displayed a statistically significant decrease in overall survival, consistent with the findings from our smaller dataset [26].

To our knowledge, there are no approved protocols in clinical practice that directly target the SNAI1 or ZEB1 pathways in pancreatic cancer. Recent study by Song L et al has demonstrated metformin increases levels of liver kinase B1 (LKB1), which is one of the key players suppresing Snail1 protein. However all clinical investigations are in the very early stage [27].

## Conclusions

In conclusion, the expression of EMT-TFs is significantly altered in PDAC, with SNAI1, SNAI2, ZEB1, and TWIST showing higher mRNA and protein levels. Our results suggest that EMT-TFs, particularly the interplay between SNAI1 and ZEB1 is associated with tumor progression, metastasis, and poor survival.

## Limitations of the study

Rarity of pancreatic cancer cases suitable for upfront surgery and the requirement for meticulous tissue collection have restricted the sample size. Patients with borderline resectable tumors who underwent neoadjuvantchemotherapy were excluded from the analysis to prevent potential distortion of the EMT panel. Our study was not set to assess the functional validation and was validated externally. Furthermore, the study focused exclusively on epithelial tumor cell EMT-TF expression without assessing stromal or immune microenvironment interactions, so these aspects should serve as directions for further investigations.

## Supporting information

S1 Raw image Raw images (blot). (PDF)
