# Supplementary figures and images for: Overexpression of EMT-related transcription factors SNAI1 and ZEB1 is associated with more aggressive clinicopathological features of pancreatic cancer
Source: PLoS One. 2026 Jan 2;21(1):e0339964. doi: 10.1371/journal.pone.0339964 (PMC12758774; doi:10.1371/journal.pone.0339964)

EMT 1A Snail/SLUG

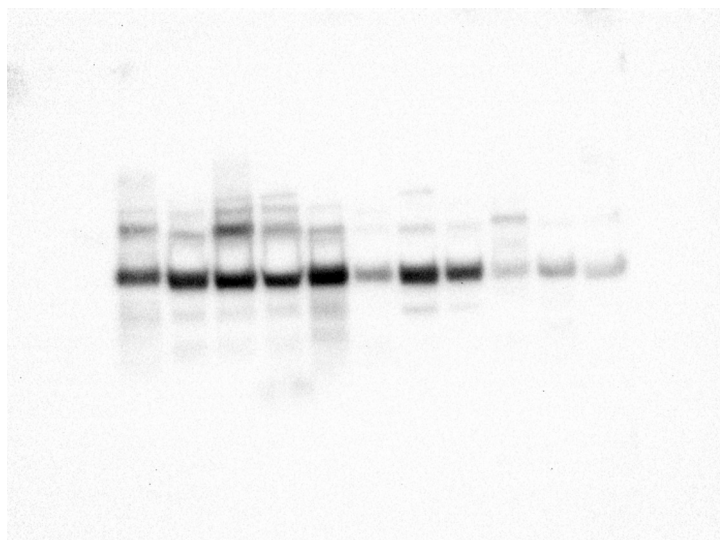

EMT 1A Zeb1 300s

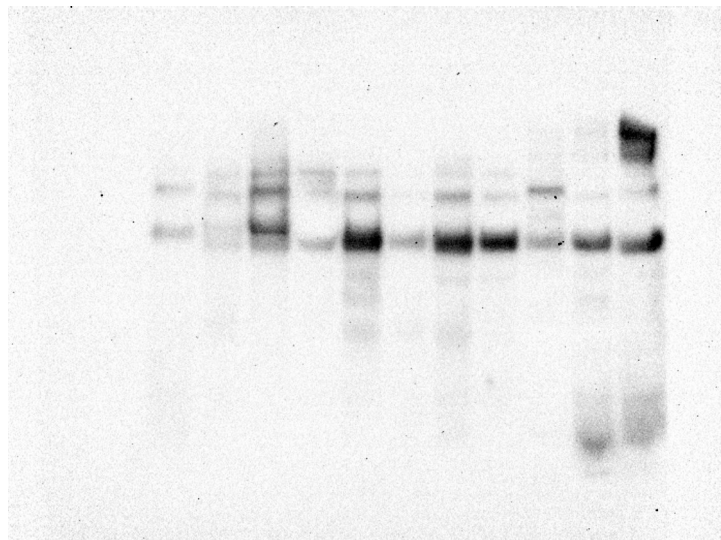

EMT 3A Zeb2 300s

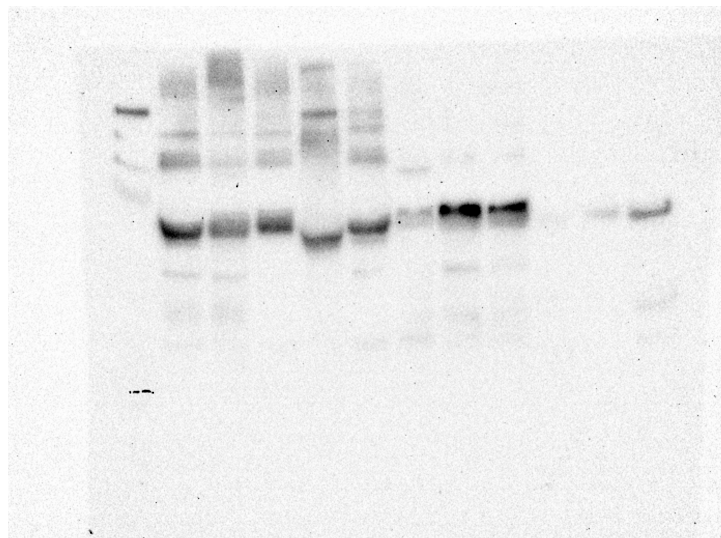

EMT 3A Twist 180s

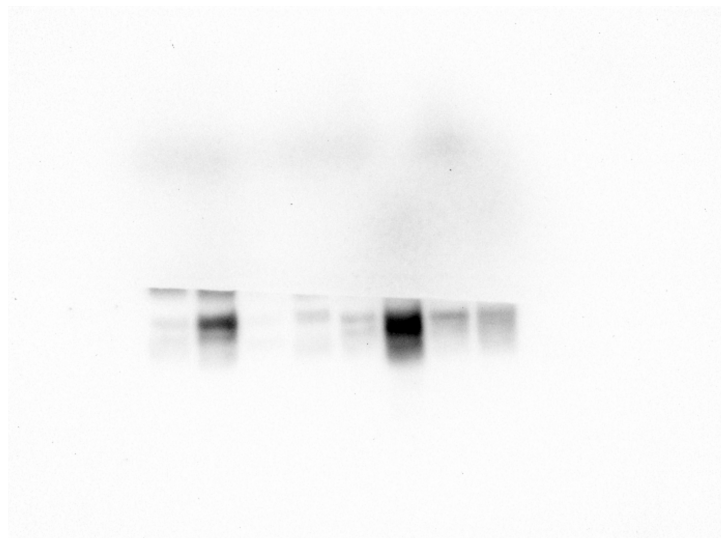

GAPDH 180s

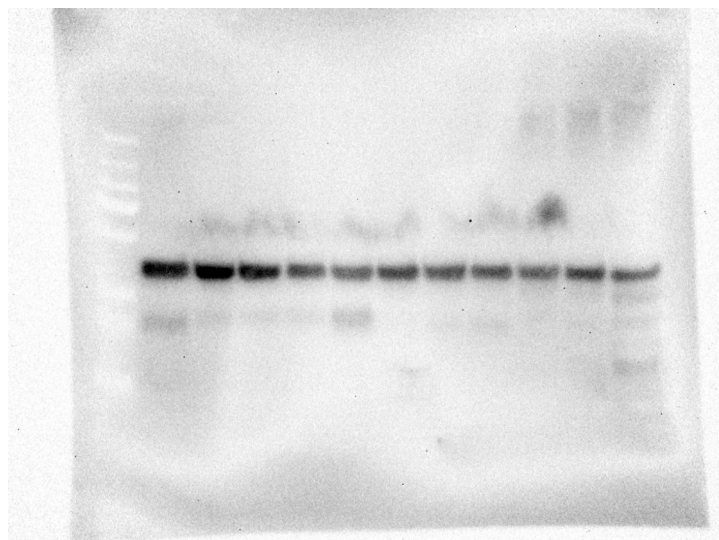

Supplement: S1 Raw image — (PDF) [file pone.0339964.s001.pdf]
